# Supplementary material for: Content-rich biological network constructed by mining PubMed abstracts
Source: BMC Bioinformatics. 2004 Oct 8;5:147. doi: 10.1186/1471-2105-5-147 (PMC528731; doi:10.1186/1471-2105-5-147)
Supplement: Additional File 5 — The original Chilibot query results of the term "long-term potentiation (LTP)" and 22 other terms, limiting the latest references analyzed to the years 1990, 1995, 2000, and 2004. [file 1471-2105-5-147-S5.bz2 › chilibotAdditionalFile5/ltp2004/html/PLC_AMPA.html]

 


 **PLC** and **AMPA** 
  
Found 6 abstracts in PubMed,  **6 abstracts were retrieved and analyzed**.  


---

 Search Google  |
 PDF files only 
|  EDU domain only 

---

**Interactive relationship** (e.g. stimulation, inhibition, etc)

- Recently, we reported that  **AMPA**  receptor agonists increased 45Ca2 uptake and phospholipase C  **PLC**  activity.  Ref: 10095077 Brain Res Mol Brain Res, 1999
- All together, these results indicate that MAPK is implicated in the transmission of  **AMPA**  signaling to the nucleus and requires extracellular calcium, and  **PLC**  PKC activation.  Ref: 10095077 Brain Res Mol Brain Res, 1999
- These results indicate that in developing cortical neurons, activation of  **AMPA**  KA receptors by KA may induce expression, followed by activation of TrkA via  **PLC**  signaling and intracellular calcium elevation and hence increase reception of NGF on KA challenged neurons.  Ref: 10820201 J Neurochem, 2000
- **PLC**  from C. perfringens produced an increased affinity of the quisqualate DL alpha amino 3 hydroxy 5 methylisoxazole 4 propionic acid  **AMPA**  receptor for its ligand.  Ref: 2154675 Mol Pharmacol, 1990
- In contrast to  **PLC**  from C. perfringens, phosphatidylinositol specific  **PLC**  treatment did not detectably modify the binding properties of the quisqualate  **AMPA**  receptor or the NMDA receptor channel.  Ref: 2154675 Mol Pharmacol, 1990

**Parallel relationship** (e.g. studied together, co-existance, homology, etc.)

- This may explain increased KCl and  **AMPA**  induced InsP1 accumulation whereas receptor coupled  **PLC**  activation is less affected.  Ref: 8242388 Brain Res, 1993
- A study of polyphosphoinositide turnover showed that KA stimulated phospholipase C  **PLC**  activity was directly triggered by the  **AMPA**  KA receptor activity, but not by the activity of TrkA or other excitatory amino acid receptor subtypes.  Ref: 10820201 J Neurochem, 2000
